# Supplementary material for: Causal Relationship between Aging and Anorexia Nervosa: A White-Matter-Microstructure-Mediated Mendelian Randomization Analysis
Source: Biomedicines. 2024 Aug 16;12(8):1874. doi: 10.3390/biomedicines12081874 (PMC11351342; doi:10.3390/biomedicines12081874)
Supplement: Supplementary file 1 [file biomedicines-12-01874-s001.zip › Supplementary Figure/Figure S8/Leaveoneout plot of MR analysis for TL to ubm-b-1928.pdf]

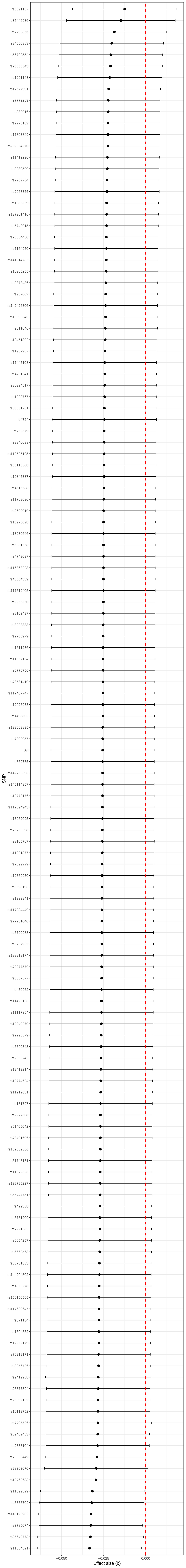

rs3891167
rs35446936
rs7790856
rs34550383
rs56799554
rs76065543
rs1291143
rs17677991
rs7772289
rs939916
rs2276182
rs17803849
rs202034370
rs11412296
rs2230590
rs2282764
rs2967355
rs1985369
rs137901416
rs5742915
rs75664430
rs7164950
rs141214782
rs10905255
rs9878436
rs932902
rs142426306
rs10805346
rs611646
rs12451892
rs1957937
rs17445108
rs4731541
rs80324517
rs1023767
rs56061761
rs4724
rs762679
rs9940099
rs113525195
rs80116508
rs10845387
rs4616688
rs11769630
rs9600019
rs16978028
rs13230646
rs6881568
rs4743037
rs116863223
rs45604339
rs117512405
rs9955360
rs8102497
rs3093888
rs2763979
rs1611236
rs11557154
rs6776756
rs73581419
rs117407747
rs12925933
rs6498805
rs139669835
rs7209057
All
rs869785
rs142730696
rs145114957
rs10773176
rs112394943
rs13062095
rs73730598
rs8105767
rs11991877
rs7099229
rs12369950
rs9398196
rs1332941
rs117034449
rs77231040
rs6790988
rs3767952
rs188918174
rs79977579
rs6587577
rs450962
rs11426156
rs11117354
rs10840270
rs2293579
rs6590343
rs2538745
rs12412214
rs10774624
rs11212631
rs131797
rs2977608
rs61405042
rs78491606
rs182059586
rs61748181
rs11579626
rs139795227
rs55747751
rs429358
rs6751209
rs7221585
rs6054257
rs6669563
rs66731853
rs144204502
rs4530278
rs150150565
rs117630647
rs871134
rs41304832
rs12932179
rs76219171
rs2056726
rs9419958
rs28577594
rs28502153
rs10112752
rs7705526
rs59409453
rs2555104
rs76666449
rs28363070
rs10768683
rs11699829
rs6536702
rs143190905
rs3785074
rs35640778
rs11584821
SNP
-0.050
-0.025
0.000
Effect size (b)
